# SOY3DSEG: A high-precision universal point cloud segmentation model for soybean full growth period based on improved point transformer

**DOI:** 10.1016/j.plaphe.2026.100167

**Published:** 2026-01-10

**Authors:** Jiarui Niu, Junyao Tian, He Zhang, Hansong Zhang, Zhanguo Zhang, Dawei Xin, Qingshan Chen, Rongsheng Zhu

**Affiliations:** a College of Engineering, Northeast Agricultural University, Harbin, 150030, Heilongjiang, China; b College of Arts and Sciences, Northeast Agricultural University, Harbin, 150030, Heilongjiang, China; c School of Public Administration & Law, Northeast Agricultural University, Harbin, 150030, Heilongjiang, China; d College of Agriculture, Northeast Agricultural University, Harbin, 150030, Heilongjiang, China; e National Key Laboratory of Smart Farm Technologies and Systems, Harbin, 150030, China

**Keywords:** 3D point-cloud analysis, Soybean full-growth-cycle point cloud, Dynamic multi-stage sampling strategy, High-accuracy semantic segmentation, Cross-crop transferability

## Abstract

Three-dimensional (3D) reconstruction technologies for crops are of significant importance in the context of smart breeding and precision agriculture, as they enable accurate characterization of crop spatial architecture and developmental dynamics. Such capabilities provide essential phenotypic information for the rapid selection of breeding materials and informed agronomic decision-making. A critical requirement for the practical application of crop 3D models is high-accuracy organ-level segmentation. However, the absence of a stage-universal segmentation framework capable of operating across complete soybean growth cycle remains a major bottleneck hindering progress in this field. To address this issue, we propose SOY3DSEG—a high-precision framework based on an improved Point Transformer, designed to support the full developmental spectrum of soybean (V1–R7). The framework incorporates a novel down sampling strategy termed Dynamic Multi-Stage Sampling Strategy (DMSS), alongside multi-scale feature enhancement and a local geometry-aware attention mechanism, enhancing segmentation accuracy and efficiency. Performance evaluations across 12 consecutive soybean growth stages (V1 to R7) indicate that SOY3DSEG achieved an average mean Intersection-over-Union (mIoU) of 93.34 % for stem-leaf segmentation—surpassing RandLA-Net, BAAF-Net, PointNet++, and PointConv by over 30 %, and outperforming the baseline Point Transformer by 14.18 %. A moderate accuracy decline appears at R6–R7 due to dense canopies and strong occlusion, yet SOY3DSEG retains clear superiority over the baseline Point Transformer, demonstrating robustness under complex morphology. In cross-crop transfer tests limited to early seedling stages of maize and tomato, the model achieves an mIoU of approximately 99 %, indicating strong early-stage transferability while mature-stage generalization across species remains open for future study. SOY3DSEG thus provides a stage-robust and scalable solution for full-cycle soybean phenotyping and growth monitoring, contributing to precision agricultural practice.

## 1 Introduction

Three-dimensional (3D) point-cloud technology, which can precisely capture the spatial structure and developmental dynamics of crops, shows vast potential in precision agriculture across the entire growth cycle [[1], [2], [3]]. A recent LiDAR-based plant phenomics survey underscores how 3D sensing is reshaping breeding and crop-management research frontiers [4], confirming its strategic value for comprehensive, full-season phenotypic analysis. The primary objective of crop phenotyping is to elucidate complex traits arising from genotype–environment interactions, a goal that demands data spanning all growth stages to inform optimized breeding strategies and precision farm management [5,6]. Conventional phenotyping methods—relying on manual observations at the plot or individual-plant scale—are labor-intensive, time-consuming, and inadequate for the high-throughput, high-precision data acquisition needs of modern agriculture [7,8]. Recent advances in non-contact imaging sensors have made plant phenotypic data collection increasingly automated and digital, enabling significant progress in organ-level analysis of leaves, stems, and fruits [9,10]. Crucially, however, existing imaging models often fail to generalize across the full life cycle of crops. For instance, Li et al. [11] introduced PlantNet, an end-to-end network capable of segmenting multiple crop seedlings, demonstrating the feasibility of cross-crop segmentation models. Yet PlantNet's performance deteriorated at later growth stages, highlighting a pressing need for a truly universal point-cloud segmentation model that remains effective throughout a plant's entire developmental course.

Conventional two-dimensional (2D) image analysis methods are inherently limited by viewpoint constraints, sensitivity to illumination, and frequent organ occlusion, which prevent them from accurately representing the 3D structural complexity of crops [12,13]. As a result, 2D methods have been largely confined to morphologically simple or early-stage plants—such as *Arabidopsis thaliana*, tobacco, or young monocot species like wheat and maize—and struggle with complex canopies at advanced growth stages. In crops with dense, overlapping foliage, these traditional approaches become impractical. By contrast, 3D point clouds provide much richer spatial–geometric information, alleviating problems of perspective and occlusion and better capturing the true architecture of crops [[13], [14], [15]]. This makes 3D approaches inherently more scalable across growth stages, as they maintain fidelity even as plant structures become larger and more complex. For large-scale, dynamic monitoring and phenotypic trait extraction throughout the growing season, point-cloud technology thus opens new opportunities for precision agriculture [12,16].

The rapid development of 3D sensing technologies—such as LiDAR, structured-light scanners, and time-of-flight cameras—has dramatically improved the efficiency and accuracy of point-cloud acquisition, providing robust support for continuous, full-season crop monitoring [14,17]. However, most existing point-cloud segmentation methods still rely on hand-crafted geometric features (e.g. octree partitions or normal-based metrics) that are ill-suited to the increasingly complex plant architectures observed as crops mature. In practice, these traditional methods lack the adaptability needed for segmentation across different growth stages and canopy complexities. In contrast, recent advances in deep learning, particularly neural network–based models, have led to notable improvements in segmentation accuracy and generalization capability [14,18,19]. These data-driven approaches pave the way for generic models that can handle diverse crops and developmental stages by learning rich, multi-scale feature representations. Early examples include CNN-based extraction of maize organ structures from depth images [20] and Faster R-CNN–driven segmentation of individual maize plants from terrestrial LiDAR data [21]. While pioneering, such approaches still depend on 2D image projections or bounding-box detections, leaving fine-grained, point-level organ labeling in dense 3D canopies insufficiently addressed. Consequently, a gap remains in developing a unified 3D segmentation framework that can operate directly on point clouds and remain robust from seedling stages to full canopy closure.

Even with these advances, segmentation in agricultural point clouds continues to pose significant challenges. The scarcity of large, high-quality annotated 3D plant datasets hinders effective training and evaluation of models [22]. Moreover, many existing models generalize poorly when applied across different crop species or across multiple growth stages [17], limiting their practical utility for full-season phenotyping. Finally, dense and noisy point clouds from field scans lead to bottlenecks in computational efficiency and real-time processing [14,18]. Overcoming these obstacles is essential to achieve a single segmentation approach that remains accurate and efficient throughout the crop life cycle. To tackle these challenges, we developed SOY3DSEG, a plant point-cloud segmentation framework designed to provide stage-universal performance across the soybean growth cycle. The framework integrates a Dynamic Multi-Stage Sampling Strategy (DMSS) strategy with an improved, geometry-aware multi-scale Point Transformer, achieving robust performance from early vegetative to late canopy stages.

1. Dynamic Multi-stage Sampling Strategy (DMSS): We implemented a two-step progressive down-sampling scheme (random sampling followed by farthest-point sampling) that adaptively determines an optimal sampling ratio based on each plant's bounding-box volume (indicative of plant size/growth stage) and local point-density variation. This dynamic sampling balances computational efficiency with the retention of critical features, ensuring that fine details of larger, later-stage plants are preserved without overwhelming the model.
2. We enhanced the Point Transformer architecture by embedding geometry-aware attention mechanisms that distinguish stems from leaves and cope with overlapping foliage. By encoding multi-scale contextual information alongside local geometric cues, the network accommodates the diverse organ sizes and complex spatial arrangements that arise throughout plant development, thereby improving segmentation at challenging stem–leaf boundaries and within densely overlapped canopies.
3. We conducted comprehensive experiments on soybean covering 12 stages (V1–R7) and early seedling datasets of maize and tomato to evaluate cross-species transfer. The results demonstrate stage-robust performance within soybean and strong early-stage transferability across species.

SOY3DSEG consistently outperforms current state-of-the-art stem–leaf segmentation models across soybean stages V1–R7 and achieves an average mIoU of 93.34 % on soybean and ≈99 % (98.0–99.8 %) on maize and tomato seedlings. The resulting lightweight, adaptive framework enables high-throughput phenotypic analysis and supports real-time crop monitoring, laying the groundwork for broader application of 3D point-cloud technologies in precision agriculture.

## 2 Materials and methods

**Fig. 1:**
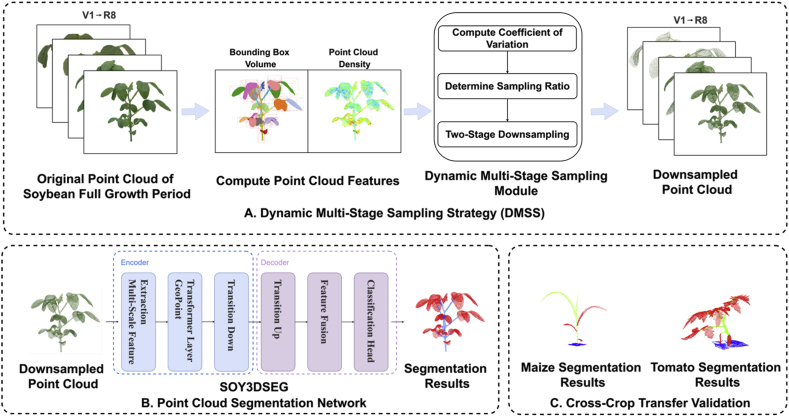
**Overview of the SOY3DSEG workflow. (A) Dynamic Multi-Stage Sampling Strategy (DMSS)**: For each soybean point cloud spanning growth stages V1–R7, the algorithm computes bounding-box volume and point-cloud density, derives the coefficient of variation, and adaptively sets a two-stage down-sampling ratio to generate a structurally faithful yet lightweight point set.**(B) SOY3DSEG point-cloud segmentation network:** An encoder–decoder architecture first extracts multi-scale geometric features with a geometry-aware Transformer and Transition Down layers, then restores resolution through Transition Up and feature-fusion blocks, finally classifying individual points into stem and leaf categories.**(C) Cross-crop transfer validation:** The trained model is applied—without additional fine-tuning—to maize and tomato datasets, producing accurate organ-level segmentations and demonstrating strong cross-species generalization.

Fig. 1 illustrates the overall architecture of the proposed framework. Raw soybean point cloud data, covering the full-growth-cycle, is first processed to compute features such as bounding box volume and point density. This information is passed to the DMSS module, where it guides the selection of sampling ratios. A two-step downsampling process is then applied to produce a reduced point cloud. The sampled data is subsequently processed through multi-scale feature extraction and a Point Transformer network, which perform segmentation and generate stem and leaf classification results. The figure also presents cross-crop transfer tests on maize and tomato, confirming the method's effectiveness across varying crop structures.

### 2.1 Point-cloud datasets collected using high-throughput methods

In the present study, two 3D point-cloud datasets were utilized: Soybean-MVS and Pheno4D, which were collected using high-throughput methods.

**Fig. 2:**
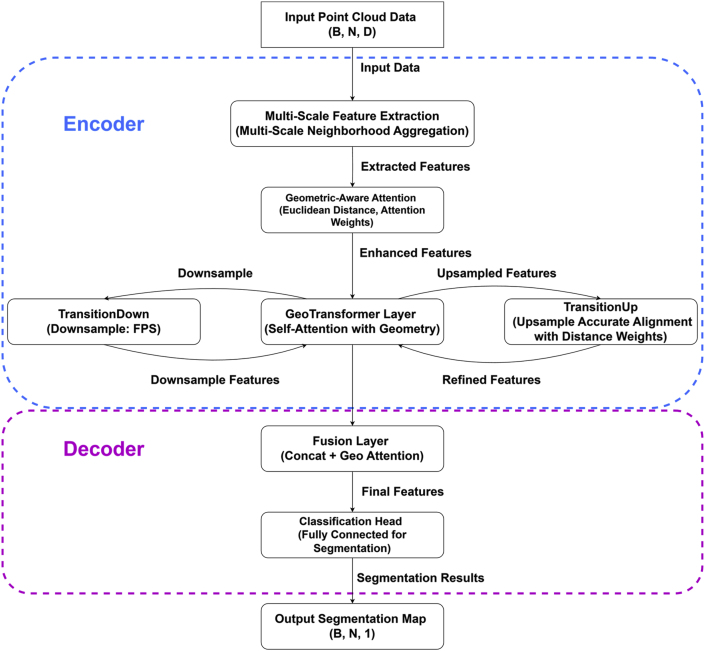
Overall architecture of the proposed SOY3DSEG network for full-growth-cycle soybean point-cloud semantic segmentation. The encoder first applies multi-scale neighborhood aggregation to the input point cloud, followed by a geometry-aware attention module and a geometry-aware attention to refine features on the down-sampled points. TransitionDown uses farthest point sampling (FPS) for hierarchical down-sampling, while TransitionUp performs distance-weighted up-sampling to accurately align coarse features back to dense points and build an encoder-side feature pyramid. The decoder fuses the encoder features through a fusion layer (concatenation and geometry-aware attention) and a fully connected classification head to produce the final per-point segmentation map.

The Soybean-MVS dataset was designed for plant phenotyping and organ-level segmentation. Soybean-MVS covers five soybean cultivars across twelve key growth stages—vegetative V1 to reproductive R7—recorded during 2018–2019. The dataset includes 102 plant samples, each represented by high-density point clouds containing millions of points, with spatial resolution finer than 0.2 mm. This level of detail enables precise visualization of morphological features such as leaf texture and stem curvature, providing comprehensive documentation of soybean development from seedling emergence (V1) to physiological maturity (R7). Fig. 2 illustrates morphological changes across growth stages. All points were manually annotated by expert annotators as either leaf or stem. The dataset's full-season coverage makes it particularly well-suited for investigating organ-level growth dynamics and for evaluating model generalization across developmental stages [23]. Further details regarding the software, annotation process, genotypes, and growth conditions can be found in the related paper. Fig. S1(see Supplementary Materials) now shows only these Soybean-MVS samples, highlighting the progressive architectural changes of a single plant throughout its life cycle.

The Pheno4D dataset supplies high-precision point clouds of maize and tomato for cross-crop studies, collected using high-throughput methods. In its tomato subset, a laser-triangulation scanner captured seven plants over 20 consecutive days, producing 140 point-cloud samples; every leaf carried an individual instance label, enabling leaf-level tracking and phenotypic parameter extraction [24].

By jointly leveraging these two datasets, the proposed DMSS and SOY3DSEG architecture was evaluated across multiple crop species and growth stages, thereby demonstrating the model's generalization capability and robustness.

### 2.2 Point-cloud down-sampling algorithms

Point-cloud down-sampling is a common technique for handling large-scale point clouds, aiming to reduce computational cost while keeping essential information. Typical methods are random sampling (RS) and farthest-point sampling (FPS).

RS reduces data size by randomly selecting a subset of points. It is computationally efficient and suitable for large datasets, but in complex crop clouds—where stems and leaves overlap—important geometric features may be lost, lowering training accuracy [25,26].

Farthest-point sampling (FPS) selects points iteratively based on maximal distance from the previously sampled set, resulting in a more uniform spatial distribution and improved retention of geometric structures such as stems, leaves, and pods [27,28]. However, the computational cost of FPS increases with the size of the point cloud, leading to extended runtimes when applied to large-scale datasets [[29], [30], [31]].

Moreover, fixed-ratio sampling strategies—whether based on random sampling (RS) or FPS—struggle to adapt to the morphological variability observed across different growth stages, often resulting in suboptimal preservation of salient features [9,25].

In this section, we describe the proposed framework for semantic segmentation of full-growth-cycle soybean point clouds. The pipeline consists of three key components: (i) a Dynamic Multi-Stage Sampling Strategy (DMSS) that adaptively selects informative points according to spatial sparsity and local structural complexity, (ii) a baseline Point Transformer backbone that provides a strong self-attention-based representation, and (iii) the SOY3DSEG network, which introduces multi-scale neighborhood aggregation and geometry-aware attention together with an encoder-side feature pyramid. These components are designed to work together to preserve critical soybean structures, enhance local geometric detail, and improve segmentation robustness across growth stages.

### 2.3 Dynamic Multi-Stage Sampling Strategy (DMSS)

#### 2.3.1 Method framework

Dynamic Multi-Stage Sampling Strategy (DMSS) is the first key component of our method. It is designed to overcome the limitations of fixed-ratio sampling in plant point clouds with highly non-uniform density, where a globally fixed budget may (i) discard critical structures in locally sparse but structurally important regions (feature loss) and (ii) preserve excessive redundant points in over-dense regions (computational redundancy). DMSS adopts an adaptive two-stage sampling process that considers both the global spatial distribution and the local point density of the point cloud, thereby dynamically optimizing the sampling ratio in response to structural complexity. Compared with fixed-ratio sampling, the core difference of DMSS is that the sampling budget is conditioned on the point cloud's structural complexity and density variability rather than being manually preset as a constant.

In the first stage, coarse random down-sampling retains 10 % of the original points to reduce data volume. In the second stage, fine dynamic FPS sampling is performed on the coarsely retained point set from the first stage (i.e., the 10 % downsampled points), rather than directly on the original point cloud. Specifically, it applies farthest point sampling with a CV-based joint criterion of voxel occupancy variation and KNN density variation, and adaptively adjusts the FPS ratio within a predefined bounded range (overall retained ratio w.r.t. the original point cloud is clamped to [2 %, 8 %]) to better preserve critical structural features.

The Stage-2 output serves as the support point set for subsequent multi-scale neighborhood aggregation in the encoder–decoder pipeline.

#### 2.3.2 Dynamic weight calculation

To make the volume–density joint criterion explicit and reproducible, we quantify the structural complexity of a point cloud using two complementary variability measures.

(1) Volume variation: we voxelize the point cloud and compute the coefficient of variation (CV) of the number of points per voxel, reflecting how unevenly the point cloud occupies the 3D space (global spread/occupancy variation).
(2) Density variation: we compute the KNN-based local density for each point and then calculate its CV, characterizing local crowding/non-uniformity caused by foliage clustering and occlusion.

We combine the two measures into a single joint criterion via a weighted sum, $C_{joint}=\lambda \;\cdot \;CV_{voxel}+\left(1-\lambda \right)\cdot \;CV_{knn}$, where λ balances the emphasis between global volume distribution and local density non-uniformity (λ is fixed in our experiments to provide a stable trade-off across growth stages).

We choose volume and density because they capture two distinct, plant-specific sources of sampling difficulty: global structural spread changes with growth, and local density extremes arise from leaf clustering and self-occlusion; using both enables DMSS to allocate sampling budget more appropriately than using either one alone.

To measure spatial sparsity and local complexity, the following statistics are defined:

Spatial sparsity weight: After dividing the point cloud's bounding box into uniform voxels (with an edge length of l = 5 cm), the coefficient of variation of the point count within each voxel is calculated:

(1) $$\begin{array}{c} CV_{v}=\frac{\sigma_{N}}{\mu_{N}} \end{array}$$

(2) $$\begin{array}{c} w_{v}=\frac{1}{1+CV_{v}} \end{array}$$

Here, μN and σN represent the mean and standard deviation of point counts across voxels, respectively. A larger CVv indicates a more uneven spatial distribution, requiring a lower sampling ratio to preserve features in sparse regions.

Local complexity weight: The coefficient of variation of k-nearest neighbor (k = 50) point densities is computed as:

(3) $$\begin{array}{c} CV_{d}=\frac{\sigma_{\rho }}{\mu_{\rho }} \end{array}$$

(4) $$\begin{array}{c} w_{d}=\frac{CV_{d}}{CV_{v}+CV_{d}} \end{array}$$

Here, $\rho_{i}=\frac{k}{\sum_{j=1}^{k}|p_{i}-p_{j}{|}_{2}}$ represents the local density of point i. A higher ${\;CV}_{d}$ indicates greater local structural complexity (e.g., overlapping leaves), and thus requires a higher sampling ratio.

Dynamic sampling ratio:Based on the combined weights, the final FPS sampling ratio is computed as:

(5) $$\begin{array}{c} r_{\text{FPS}}=r_{\text{base}}\cdot \left(w_{v}+\lambda w_{d}\right) \end{array}$$

The complete implementation is detailed in Algorithm S1(see Supplementary Materials).

### 2.4 Baseline model: point transformer

#### 2.4.1 Network architecture

The Point Transformer Layer is the core component of the baseline model. It is designed to process point-cloud data using a self-attention mechanism, allowing each point to learn enhanced local features[32]. The process consists of the following key steps:

1) Compute query (q), key (k), and value (v) via linear transformations:

Equation:

(6) $$\begin{array}{c} q=W_{q}\cdot x,k=W_{k}\cdot x,v=W_{v}\cdot x \end{array}$$

where $W_{q}$, $W_{k}$, and $W_{v}$ are linear weight matrices used for transformation, and x is the input feature vector of a point. The goal of this step is to generate corresponding queries, keys, and values for each point to be used in the subsequent attention computation.

2) Compute attention weights between points. The attention weights are obtained by normalizing the dot product between queries and keys:

Equation:

(7) $$\begin{array}{c} attention\_weights=Softmax\left(q\cdot k^{T}\right) \end{array}$$

3) Compute new feature representations through weighted summation:

Equation:

(8) $$\begin{array}{c} \hat{x}=attention\_weights\cdot v \end{array}$$

This approach enables the model to integrate each point's features with those of its spatial neighbors, effectively capturing both local and global structural information.

The baseline model employs an attention mechanism to assign weights to local and global features, generating feature representations for each point. However, it does not incorporate explicit multi-scale feature extraction or geometry-aware enhancements. As a result, while the network performs effectively on relatively simple point clouds, it may experience performance degradation in more complex scenarios due to insufficient capture of fine-grained local geometric details. In particular, its limited capacity to represent multi-scale structures leads to diminished accuracy on full-growth-cycle soybean point clouds, especially in regions with substantial geometric variation such as stem–leaf junctions. These limitations motivate the design of our SOY3DSEG network, which explicitly models multi-scale neighborhoods and geometry-aware attention to better handle complex agricultural point clouds.

### 2.5 SOY3DSEG optimized for full-growth-cycle soybean point clouds

Unlike indoor point cloud benchmarks such as S3DIS, soybean plant point clouds exhibit (i) strong and growth-stage-dependent non-uniform density, (ii) non-rigid organ geometries with thin leaves and slender stems, and (iii) heavy self-occlusion in dense canopies at late stages. These properties make a direct use of standard indoor-scene processing (e.g., fixed-ratio sampling and single-scale neighborhood settings) suboptimal for plant phenotyping.

In SOY3DSEG, we adopt a plant-oriented processing strategy while keeping the core Point Transformer operator unchanged. Specifically, (1) DMSS is applied before transformer encoding to regulate the input point distribution across sparse and dense regions; (2) multi-scale neighborhood querying is used at different layers to jointly capture fine organ boundaries (e.g., stem–leaf interfaces) and larger-scale canopy structures; and (3) geometry-aware attention explicitly incorporates relative geometric cues to better distinguish organs with similar appearance but different spatial configurations. These adaptations improve robustness across growth stages and facilitate reproduction under plant-specific point cloud characteristics.

To process full-growth-cycle soybean point clouds and improve semantic segmentation accuracy, we propose SOY3DSEG. This network incorporates three main components: (i) a multi-scale neighborhood aggregation module that captures leaf-, branch-, and plant-level structures through carefully designed ball-query radii; (ii) a geometry-aware attention mechanism that leverages point-wise geometric similarity to emphasize regions with strong local shape variation; and (iii) an encoder-side feature pyramid with distance-weighted up-sampling that refines and fuses features at multiple resolutions before they are passed to the decoder. Together, these components enhance detail capture across growth stages and enable robust segmentation under large structural variations [33,34].

Instead of using 2D convolution kernels on a regular grid, SOY3DSEG performs multi-scale neighborhood aggregation directly on the irregular point cloud. For each point i with 3D coordinates p_i and feature f_i, we construct three neighborhoods at different scales using ball query radii r∈{r1,r2,r3}. In our implementation, the ball query radii were set to r1 = 0.05, r2 = 0.10, and r3 = 0.15 (normalized by the plant height).

Equation:

(9) $$\begin{array}{c} N_{s\left(i\right)}=ball\left(p_{i},r_{s}\right),s\in \left\{1,2,3\right\} \end{array}$$

For each neighbor $j\in N_{s}\left(i\right)$, we encode the relative geometry as $\Delta p_{ij}=p_{j}-p_{i}$ and its Euclidean norm $∥\Delta p_{ij}{∥}_{2}$. These geometric terms are fed into a small MLP to obtain a learnable positional embedding $\psi_{ij}$. The message from point j to point i at scale s is then computed as $\phi_{s}\left(\left[f_{j}-f_{i};\;\psi_{ij}\right]\right)$, where $\phi_{s}\left(\cdot \right)$ is a shared MLP. Features at scale s are obtained by aggregating messages over the neighborhood:

Equation:

(10) $$\begin{array}{c} h_{i}^{s}=Agg_{j\in N_{s\left(i\right)}}\varphi_{s\left(\left[f_{j}-f_{i},\psi_{ij}\right]\right)} \end{array}$$

where Agg(·) denotes an attention-weighted summation defined below. The outputs from the three scales are concatenated to form the multi-scale feature representation.

Equation:

(11) $$\begin{array}{c} x_{i}^{multi}=Concat\left(h_{i}^{1},h_{i}^{2},h_{i}^{3}\right) \end{array}$$

To further emphasize regions with strong local geometric variation, we introduce a geometry-aware attention mechanism based on geometric similarity. For each point pair (i,j), we first compute the Euclidean distance

Equation:

(12) $$\begin{array}{c} d_{ij}={\left|\left|p_{i}-p_{j}\right|\right|}_{2} \end{array}$$

Instead of directly applying Softmax to $d_{ij}$, which would incorrectly assign larger weights to more distant points, we define an attention score $e_{ij}$ as a decreasing function of the distance, for example $e_{ij}=-d_{ij}/\sigma$, and obtain normalized attention weights

Equation:

(13) $$\begin{array}{c} a\_ij=Softmax\_j\left(e\_ij\;\right)\; \end{array}$$

so that nearer points receive larger prior weights. The geometry-aware feature of point i is then computed as a weighted sum of its neighbors

Equation:

(14) $$\begin{array}{c} x_{i}^{geo}=\Sigma_{\left\{j\in N\left(i\right)\right\}a_{ij}f_{j}} \end{array}$$

which encourages the network to focus on geometrically salient regions such as leaf margins and stem–leaf boundaries.

Finally, SOY3DSEG fuses the multi-scale and geometry-aware features by channel concatenation followed by a linear projection:

Equation:

(15) $$\begin{array}{c} x_{i}^{fused}=W_{f}\left[\;x_{i}^{multi};\;x_{i}^{geo}\right] \end{array}$$

The fused features are passed to subsequent encoder–decoder layers and a fully connected classification head to produce the final point-wise segmentation results. As illustrated in Fig. 2, the multi-scale aggregation and geometry-aware attention operate inside the encoder, where down-sampling and distance-weighted up-sampling form an encoder-side feature pyramid while preserving accurate geometric alignment between different resolutions. In this way, SOY3DSEG jointly leverages dynamic sampling, multi-scale neighborhood aggregation, and geometry-aware feature refinement to address the challenges of full-growth-cycle soybean point-cloud segmentation.

## 3 Experiments

### 3.1 Data augmentation strategies and dataset splits

To enhance the model's adaptability to complex point cloud features encountered in agricultural environments, a data augmentation framework informed by crop growth characteristics was developed. This framework addresses common challenges in field data acquisition, including viewpoint shifts, scale variations, and local occlusions. Three augmentation strategies are employed: CenterShift, RandomScale, and RandomFlip.

CenterShift applies random displacements within ±0.5 m along the three coordinate axes to the point cloud center, simulating positioning errors caused by field equipment movement, thereby enhancing the model's adaptability to samples at different spatial locations [35,36].

RandomScale, based on the dynamic change in soybean plant height from 15 cm to 120 cm during growth, performs isotropic scaling with a scale factor in the range of [0.8, 1.2], improving the model's robustness to scale variations across growth stages [37,38].

RandomFlip, grounded in the mirror symmetry of plant organs (e.g., leaves), applies random flips along the XZ plane to simulate multi-directional viewpoints and mitigate geometric feature loss caused by one-sided scanning [39,40].

This diversified augmentation strategy enhances the model's generalization capability and stability in both cross-stage and cross-crop applications, thereby establishing a robust preprocessing foundation for point cloud segmentation tasks in agricultural contexts.

For dataset splitting, a dual strategy combining spatiotemporal decoupling and cross-species generalization validation was used. The main soybean dataset was divided by year and cultivar: the training set integrates full-growth-cycle data from five cultivars (DN251–DN255) in 2018–2019, the validation set uses data from the single cultivar DN252 in 2019 (V3–R6 stages) for hyperparameter tuning, and the test set isolates full-cycle data from DN251 in 2019 to evaluate out-of-sequence extrapolation ability.

To further verify the model's generalizability in continuous growth monitoring, a temporal decoupling validation framework was constructed based on the Pheno4D dataset. The training set integrates full-sequence point clouds from multiple tomato and maize plants, while the test set uses full-sequence data from a single plant.

### 3.2 Evaluation metrics

In the present study, the mean of IoU scores for two classes (mIoU) and mean accuracy (mAcc) were adopted to evaluate the performance of each architecture. TP, TN, FP, and FN represent the true positive, true negative, false positive, and false negative counts for each class, respectively. The definitions of the semantic class IoU, per-plant accuracy, mIoU, and mAcc are as follows:

1) Accuracy: Measures the proportion of correctly classified points.

(16) $$\begin{array}{c} \text{Accuracy}=\frac{TP+TN}{TP+TN+FP+FN} \end{array}$$

2.) IoU: Overlap between predicted and true points of a class.

(17) $$\begin{array}{c} \text{IoU}=\frac{TP}{TP+FP+FN} \end{array}$$

3. mIoU: The mean of IoUs for the stem and leaf classes.

(18) $$\begin{array}{c} \text{mIoU}=\frac{{\text{IoU}}_{\text{Leaf}}+{\text{IoU}}_{\text{Stem}}}{2} \end{array}$$

4. mAcc: The mean of classification accuracies for the stem and leaf classes.

(19) $$\begin{array}{c} \text{mAcc}=\frac{{\text{Acc}}_{\text{Leaf}}+{\text{Acc}}_{\text{Stem}}}{2} \end{array}$$

### 3.3 Network training and testing

An end-to-end training strategy was adopted in this experiment using a standard deep learning framework. Combined with various data augmentation methods, the model was evaluated using the training, validation, and test sets.

During training, the AdamW optimizer was employed with an initial learning rate of 0.003, combined with a learning rate decay strategy to facilitate convergence. A batch size of 2 was used, and training was conducted on a single NVIDIA RTX 4090 GPU. To mitigate overfitting, an early stopping mechanism was implemented, terminating training when the validation loss exhibited no significant improvement over a defined period. Additionally, automatic mixed precision (AMP) training was enabled to accelerate computation and reduce memory consumption, thereby optimizing the use of computational resources.

During testing, the model was evaluated on the test set, which was not involved in training. Multiple metrics (IoU, mIoU, Acc, mAcc) were used to assess model performance across different crops and growth stages.

The hardware platform used in the present study included an NVIDIA RTX 4090 GPU (24 GB VRAM) and an AMD 9950X CPU (16 cores, 32 threads, 4.5 GHz base frequency). The software environment was PyTorch 2.1.0 with CUDA 11.8, which is sufficient for full model training on a consumer-grade system. The training schedules for different crops using SOY3DSEG are provided in the Supplementary Materials (Table S7.).

## 4 Results

### 4.1 Performance of the DMSS

In the present study, DMSS demonstrated significant advantages in processing soybean point-cloud data across different growth stages (V2, R1, R4, R7). Compared with traditional sampling methods such as Random Sampling and Farthest Point Sampling, DMSS could determine which plant point clouds required denser sampling based on volume and density metrics. This allowed the method to better preserve the structural details of complex plants under the same computational load.

#### 4.1.1 Sampling effect visualization

Figs. S2 and S3(see Supplementary Materials) compare the sampling effects of Random Sampling (RS), Farthest-Point Sampling (FPS) and the proposed Dynamic Multi-Stage Sampling Strategy (DMSS) on soybean point-cloud data.

Specifically, DMSS dynamically adjusted the sampling ratio based on the computed volume and average point density of the input cloud, allocating a greater number of points to denser regions or larger plants to better preserve structural features. For instance, during the V2 and V5 stages, when soybean plants exhibited relatively simple architectures, DMSS efficiently retained critical details with fewer sampled points. Conversely, in later stages such as R4 and R6, where plant density and structural complexity increased, the strategy allocated more points to geometrically intricate areas, including stem–leaf junctions and overlapping foliage.

By comparison, while Random Sampling and Farthest Point Sampling can spatially cover the point cloud, they failed to consider plant volume and density, and thus could not adaptively allocate points based on regional complexity. This resulted in the loss of important features in structurally complex regions.

In summary, DMSS could determine the sampling strategy by jointly considering plant volume and density, ensuring that under the same computational load, more sampling points were allocated to complex plants. This better preserved plant morphology—particularly in dense and structurally intricate areas—and outperformed traditional sampling methods.

#### 4.1.2 Sampling accuracy comparison

To evaluate how well different sampling methods preserve key points, the ISS (Intrinsic Shape Signatures) method was adopted to extract key points from the point clouds [41], and compare the accuracy of each sampling method by calculating the proportion of key points retained among all sampled points [34].

The ISS method is a keypoint extraction technique that identifies representative structural features within a point cloud based on local geometric properties [41]. It operates by analyzing the geometric configuration surrounding each point, computing attributes such as local curvature and normal direction to determine points that are most descriptive of the overall structure. By focusing on regions with distinctive local geometry, ISS effectively captures salient features critical for downstream tasks such as registration, matching, and segmentation.

In the present study, the ISS method was used to extract key points from each dataset, which were then used for subsequent comparison of different sampling methods. This approach enabled a quantitative assessment of each downsampling method's ability to preserve critical points, particularly in the context of complex agricultural point cloud data.

As shown in Table S1(see Supplementary Materials), the DMSS method achieved a higher keypoint retention ratio across all test datasets compared to the RS and FPS methods. Specifically, DMSS yielded an average keypoint retention ratio of 0.8119, significantly outperforming RS (0.7383) and FPS (0.7958). This result further demonstrates the advantage of DMSS in balancing sampling efficiency and feature preservation, particularly in retaining critical structural features when handling complex crop point-cloud data.

#### 4.1.3 Sampling efficiency comparison

DMSS not only excels in preserving structural features but also significantly improves sampling efficiency. Table S2(see Supplementary Materials) presents a comparison of sampling times for different methods across various crop datasets.

The comparison of sampling times shows that the dynamic sampling method significantly improved sampling efficiency. Particularly on the soybean and maize datasets, it demonstrated much faster performance than Farthest Point Sampling, with efficiency gains of 44.8 %, 89.6 %, and 69.2 % on the soybean, maize, and tomato datasets respectively, while also achieving better preservation of crop feature information.

### 4.2 Model performance across soybean growth stages

The experimental results show that as soybean plants grew, the complexity of point-cloud data increased. From the V1 (seedling) to the R7 (mature) stage, the density and structural complexity of point clouds rose, requiring segmentation models to be more adaptive. Throughout this progression, SOY3DSEG demonstrated exceptional stability and performance.

**Fig. 3:**
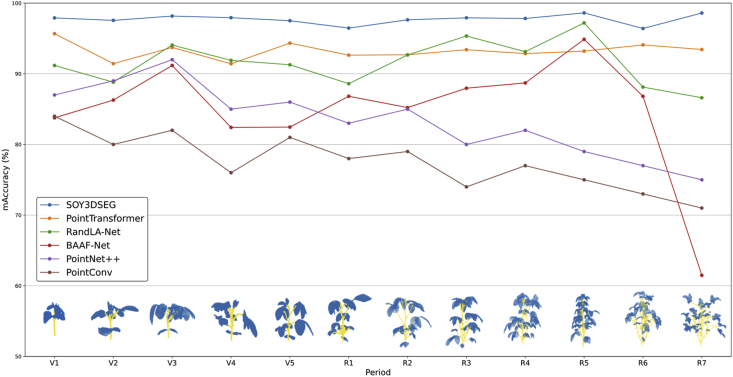
Model comparison of mean accuracy (mAcc) for stem–leaf segmentation over the entire soybean growth cycle (V1–R7). The line chart reports mAcc (%) for six networks—SOY3DSEG, Point Transformer, RandLA-Net, BAAF-Net, PointNet++, and PointConv—while the thumbnails underneath show representative SOY3DSEG outputs at each stage (blue = leaf, yellow = stem). SOY3DSEG consistently exceeds 95 % accuracy throughout all stages and shows the largest performance margin during the reproductive phase (R1–R7).

As shown in Fig. 3, SOY3DSEG maintained a consistently high accuracy across different soybean growth stages, especially during later stages like R6 and R7, where accuracy remained above 90 %. In contrast, PointConv and PointTransformer exhibited more fluctuation in these stages and failed to achieve stable performance improvements. Specifically, SOY3DSEG achieved an accuracy of 98.21 % at the R6 stage and 96.44 % at the R7 stage, both significantly higher than those of other models. This indicates that SOY3DSEG can maintain high segmentation accuracy as crop structures become increasingly complex and dense during growth. Detailed training results for all models and stages are available in Supplementary Materials Tables S3 and S4.

The learning-rate, loss, mAcc and mIoU curves for both training and validation sets follow nearly parallel trajectories and converge smoothly, demonstrating that the network's high test-set accuracy reflects true learning of stem-leaf patterns across growth stages. The training process curves are displayed in the Supplementary Materials Fig. S4.

**Fig. 4:**
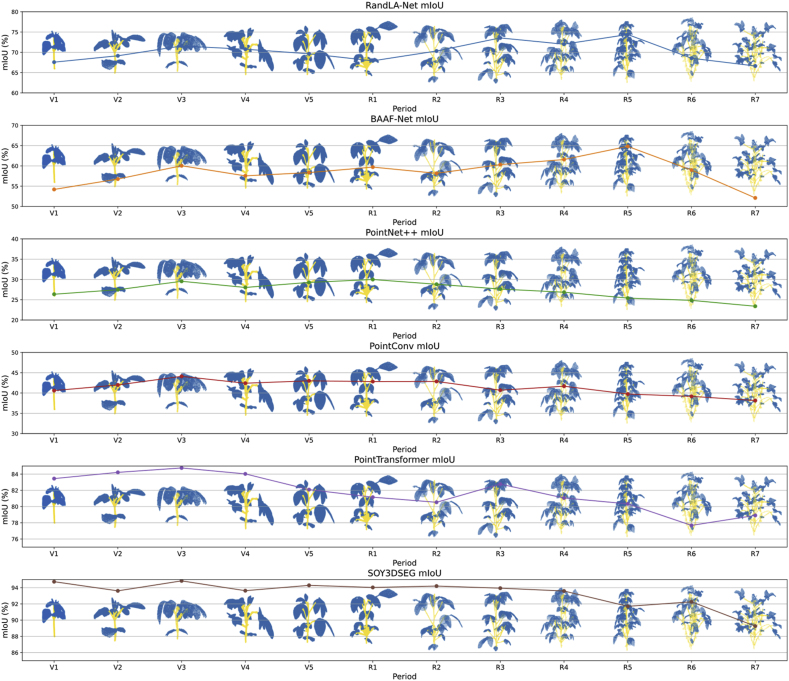
Mean Intersection-over-Union (mIoU) trends of benchmark models across the soybean growth cycle. Each subplot plots the mIoU (%) trajectory of one network—RandLA-Net, BAAF-Net, PointNet++, PointConv, Point Transformer, and SOY3DSEG—from V1 to R7, accompanied by sample segmentation snapshots (blue = leaf, yellow = stem). SOY3DSEG maintains high mIoU (≈80–93 %) across all stages and demonstrates superior robustness in the dense reproductive canopies of the later stages.

In terms of mIoU, SOY3DSEG also demonstrated a clear advantage, especially during the later growth stages of soybean (e.g., R6 and R7). As shown in Table S4 (see Supplementary Materials), SOY3DSEG's mIoU was 10 %–15 % higher than that of other models. For instance, at the R6 stage, SOY3DSEG achieved an mIoU of 91.70 %, while PointNet++ and RandLA-Net reached only 25.41 % and 64.82 %, respectively (see Fig. 4). This highlights SOY3DSEG's superior segmentation capability in overlapping regions such as stem–leaf junctions.

Moreover, SOY3DSEG consistently maintained high mIoU values across different growth stages, particularly in the later stages. In contrast, PointNet++ and RandLA-Net showed relatively steady but lower segmentation performance in later growth stages. At the V1 and V2 stages, SOY3DSEG achieved mIoU values of 94.74 and 93.61, respectively, and still maintained 89.26 at the R7 stage—all at high levels—demonstrating its robust adaptability from the seedling stage to maturity.

These results demonstrate that SOY3DSEG could effectively handle complex point cloud data while maintaining high segmentation accuracy as crop structures become increasingly intricate and dense, highlighting its strong potential for broad application in agricultural phenotyping and precision farming.

### 4.3 Cross-crop transfer performance of SOY3DSEG

This section presents the segmentation results of the SOY3DSEG framework on point cloud datasets from different crop types. Tables S5 and S6 report the mIoU and accuracy values achieved by SOY3DSEG on maize and tomato datasets at the seedling stage. These results demonstrate the model's consistent performance and robustness in cross-crop transfer tasks.

Table S5 (see Supplementary Materials) shows the segmentation results on the maize seedling dataset. Throughout the experiment, the mIoU for maize segmentation remained above 99 %, reaching a peak of 99.8 %. This indicates that SOY3DSEG maintained high segmentation accuracy when processing maize seedling data.

Table S6 (see Supplementary Materials) presents the segmentation results on the tomato seedling dataset. For tomato, the mIoU was relatively high in the early stages, peaking at 99.2 %. However, as the growth stage advanced, segmentation accuracy gradually declined, dropping to 98.3 % by day 20. This trend suggests that SOY3DSEG's segmentation accuracy experienced slight fluctuations as the tomato developed over time.

Experimental results on both datasets show that SOY3DSEG consistently achieves high mIoU and Accuracy on point-cloud data of different crops at their structurally simple seedling stages, highlighting its strong effectiveness and stability in cross-crop transfer tasks.

### 4.4 Ablation study

To further evaluate the contribution of individual components within the SOY3DSEG framework, a series of ablation experiments was conducted by systematically removing or substituting key modules. The objective was to quantify the impact of each component on both segmentation accuracy and computational efficiency. These experiments specifically assessed the roles of multi-scale feature fusion, the geometry-aware attention mechanism, and the DMSS in processing full-growth-cycle soybean point cloud data.

The ablation results (see Table 1, Table 2) illustrate how removing each module affected evaluation metrics such as mIoU and Accuracy.

**Table 1:** Effect of removing key components on classification accuracy metrics.

Evaluate metrics/Models	Full model	RS dataset	No geometry-aware attention mechanism	Remove multi-scale feature fusion
Acc (leaf)	99.43	97.12	98.52	98.21
Acc (stem)	95.57	89.45	91.85	91.48
mAcc	97.50	93.29	95.19	94.84

**Table 2:** Effect of removing key components on segmentation IoU metrics.

Evaluate metrics/Models	Full model	RS dataset	No geometry-aware attention mechanism	Remove multi-scale feature fusion
IoU (leaf)	98.27	94.56	96.78	96.35
IoU (stem)	88.41	81.33	85.12	85.73
mIoU	93.34	87.95	90.95	90.84

In the ablation experiments, removing the DMSS led to a drop in mIoU from 93.34 % to 87.95 %, highlighting the crucial role of DMSS in handling complex regions, particularly at the stem–leaf junction. Acc (stem) and Acc (leaf) also declined to varying degrees, indicating that this strategy effectively enhanced segmentation accuracy in high-density areas.

When the geometry-aware attention mechanism was removed, the model's mIoU decreased from 93.34 % to 90.95 %, accompanied by a drop in accuracy metrics. Notably, Acc (stem) fell from 95.57 % to 91.85 %, further confirming the importance of geometry-aware attention in handling fine-grained regions, especially in segmenting complex geometric structures.

When the multi-scale feature fusion module was removed, the model's mIoU dropped from 93.34 % to 90.84 %. In particular, there was a significant decline in segmentation accuracy for the leaf class, as reflected in both Acc (leaf) and IoU (leaf). These findings indicate that multi-scale feature fusion plays a critical role in managing structural diversity in crops and enhancing model adaptability across different growth stages, particularly when processing point cloud data exhibiting variations in morphology and structural density.

## 5 Discussion

### 5.1 Impact of growth stages on plant segmentation performance

Stage 1: Seedling Stage (Early Growth Phase)

**Fig. 5:**
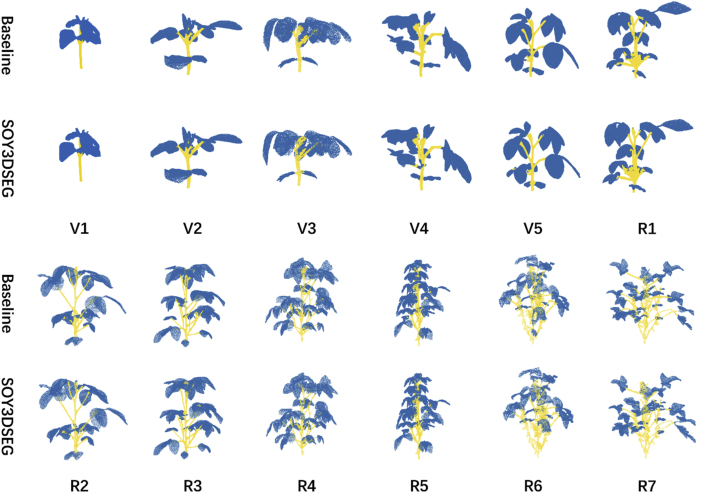
Visual comparison of 3-D point-cloud semantic segmentation for soybean throughout the entire growth cycle (V1–R7). Each column corresponds to one growth stage; leaf points are rendered in blue and stem points in yellow. Rows labeled “Baseline” show the results of a conventional backbone, whereas rows labeled “SOY3DSEG” present the outputs of the proposed method. SOY3DSEG produces cleaner leaf contours, better stem continuity, and less noise than the baseline, maintaining clear organ separation even in the densely overlapped canopies of the late reproductive stages (R6–R7).

In the seedling stage, plants are in the early phase of development, characterized by a limited number of leaves and relatively simple structural morphology. The corresponding point cloud data is small, sparse, and clearly organized, with minimal occlusion or overlap. Under these conditions, segmentation performance was high: leaves (blue) and stems (yellow) were accurately distinguished, with negligible misclassification. Overall segmentation accuracy was excellent, supported by the simplicity of the plant geometry and the absence of complex structural interference (see Fig. 5).

Stage 2: Mid-Growth Stage (Rapid Growth Phase)

During the mid-growth stage, plants exhibit increased structural complexity, characterized by a greater number of leaves, elongated stems, and expanded overall morphology. The corresponding point cloud data becomes denser, with a noticeable increase in volume and the emergence of partial leaf overlap, although the overall structure remains discernible. Segmentation performance remained robust, with the majority of leaf and stem points correctly classified. Minor classification errors may have occurred in localized regions, particularly at stem–leaf junctions; however, these misclassifications were limited in extent. While there was a slight reduction in segmentation completeness and accuracy compared to the seedling stage, the algorithm continued to demonstrate reliable performance.

Stage 3: Late Growth Stage (Structural Maturity Phase)

**Fig. 6:**
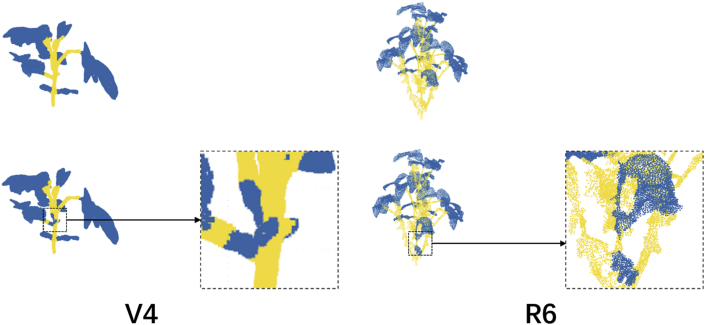
3-D point-cloud segmentation results produced by SOY3DSEG on soybean plants at growth stages V4 (vegetative) and R6 (reproductive). Leaf points are rendered in blue and stem points in yellow. The enlarged insets highlight stem–leaf junctions, demonstrating that the model preserves crisp boundaries and consistent labels even in highly overlapped regions.

During late growth, the plant exhibits a dense and highly complex structure, characterized by numerous overlapping leaves and partial occlusion of stem regions. The corresponding point cloud becomes increasingly dense. Segmentation accuracy began to decline, particularly in areas where stems and leaves were closely intertwined. Misclassifications may have occurred, with some leaf regions incorrectly labeled as stems due to geometric similarity, and vice versa. Nonetheless, the primary stem and most peripheral leaves remained correctly segmented, demonstrating the model's capacity to adapt to structurally intricate scenarios. Despite localized classification errors, the overall segmentation accuracy remained relatively high (see Fig. 6).

Stage 4: Maturity Stage (Late Full-Growth Phase)

At maturity, the plant reaches its peak complexity, with highly interwoven leaves and stems, dense point-cloud data, and severe occlusion and overlap. At this point, the algorithm faced greater challenges in handling the intricate morphology. Some inner leaves were fully occluded by outer ones, leading to incomplete segmentation. A few stems were incorrectly classified as leaves, and some leaf regions were unrecognized. Nonetheless, the main stem structure and most visible outer leaves were still accurately segmented, demonstrating the algorithm's adaptability and robustness when faced with highly complex plant structures.

Across all stages from seedling to maturity, SOY3DSEG maintained high segmentation accuracy and stability on point-cloud data at different growth phases. Although segmentation precision slightly declined with increasing structural complexity, especially due to occlusion and stem–leaf interweaving in later stages, the model consistently identified major structures, exhibiting strong robustness and cross-stage adaptability.

### 5.2 Improvement in segmentation performance by individual modules

The proposed SOY3DSEG model significantly improved segmentation accuracy and computational efficiency in analyzing soybean plant point-cloud data. By introducing the DMSS and the geometry-aware attention mechanism, SOY3DSEG demonstrated superior performance in detail capture and feature processing across different growth stages. DMSS can adaptively adjust the sampling density and range, effectively handling high-density areas and avoiding the information loss caused by over-simplification in traditional methods. This is particularly beneficial in the later growth stages of soybean (e.g., R6 and R7), where increasing structural complexity, overlapping leaves, and junctions pose major segmentation challenges. Using DMSS, SOY3DSEG improves segmentation precision in these areas, outperforming models like PointNet++ and RandLA-Net.

Meanwhile, the incorporation of the geometry-aware attention mechanism enables the model to effectively focus on critical regions, particularly in areas characterized by complex geometries such as stem–leaf junctions and overlapping leaves. By assigning greater weight to key point cloud features based on local geometric variation, this mechanism enhances the model's sensitivity to fine structural details, thereby improving segmentation performance in detail-rich regions. Ablation experiments demonstrate that the removal of this module resulted in a marked decline in segmentation accuracy, with the most pronounced performance degradation observed around stem–leaf boundaries.

Ablation experiments further verify the contribution of each SOY3DSEG module to overall performance. The results show that removing DMSS led to a substantial decline in segmentation accuracy in complex areas, particularly overlapping leaves and stem–leaf junctions. DMSS can adjust the sampling ratio based on point-cloud density, ensuring higher sampling in dense areas and preserving key features, avoiding the accuracy loss common in traditional sampling methods. The removal of the geometry-aware attention mechanism significantly impairs the model's performance in fine-detail regions, particularly at structural boundaries, resulting in less distinct and less accurate segmentation. This mechanism plays a critical role in guiding the model's attention toward important local features, thereby enhancing segmentation quality in geometrically complex areas. Further, ablation of the multi-scale feature fusion module leads to unstable performance when processing point clouds with varying spatial scales, especially across different growth stages. The multi-scale feature fusion component enables the model to adapt to dynamic changes in plant morphology and structural density, ensuring consistent and high segmentation accuracy across diverse plant structures and developmental phases.

These experiments demonstrate that every module in SOY3DSEG plays a critical role in enhancing segmentation accuracy and detail capture in soybean point-cloud data. Removing any core module significantly affects model performance in complex and fine-detail regions, further confirming their importance in improving overall model effectiveness.

### 5.3 SOY3DSEG's strong performance in cross-crop transfer tests

This section presents the segmentation results of SOY3DSEG on cross-crop point-cloud datasets, concentrating on seedling-stage **maize** and **tomato**. Deploying the model on these non-soybean species highlights its capacity to cope with intricate plant architectures, especially at stem–leaf junctions and within overlapping foliage. Quantitatively, SOY3DSEG achieves higher mIoU and mAcc than conventional baselines on both datasets.

**Fig. 7:**
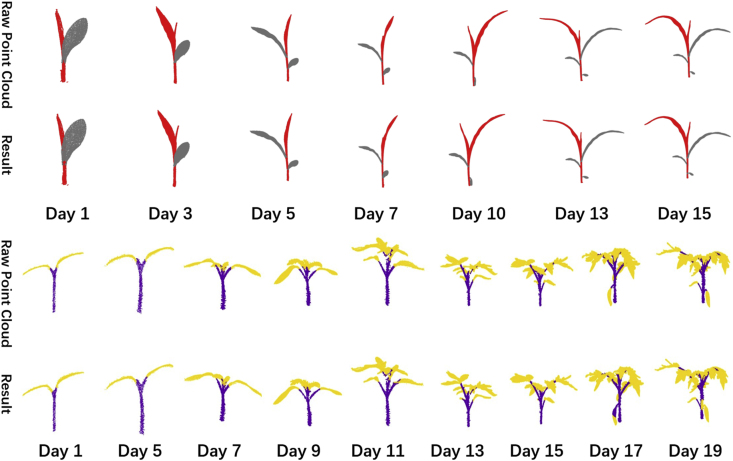
Time-lapse semantic segmentation on two crops. **Top block:** maize seedling tracked from Day 1 to Day 15 (columns). **Bottom block:** tomato seedling tracked from Day 1 to Day 19. In each block the first row shows the raw point-cloud frames and the second row shows the SOY3DSEG outputs: stem points are coloured red (maize) or purple (tomato) and leaf points grey (maize) or yellow (tomato). The model maintains clear organ boundaries and connectivity throughout the entire growth sequence.

Fig. 7 **(upper block)** displays the maize sequence collected from **Day 1 to Day 15**. SOY3DSEG consistently separates the main stem (red) from the leaves (grey), maintaining clear organ boundaries throughout rapid elongation. The segmentation is stable over time, and misclassifications along narrow stem–leaf borders are largely avoided.

Fig. 7 **(lower block)** shows the tomato sequence recorded from **Day 1 to Day 19**. Tomato point clouds are more challenging, with denser and heavily overlapping leaves. Even under this structural clutter, SOY3DSEG reliably extracts stems (purple) and leaves (yellow), preserving leaf integrity and preventing label bleeding between occluded surfaces.

These experiments demonstrate that SOY3DSEG transfers well to crops with contrasting morphologies and growth dynamics, underscoring its adaptability and robustness. Although the current evaluation is limited to seedling stages, future work will examine performance across complete growth cycles and additional species to further validate the model's generalisation ability.

### 5.4 Limitations and future directions

Although SOY3DSEG demonstrates strong performance across multiple crop types and growth stages, several limitations remain. A primary constraint is the reliance on large volumes of annotated point cloud data for training, which typically requires strong supervision and involves substantial manual labeling effort. While this ensures accurate supervision signals, the associated labor and time costs can be prohibitive. To address this issue, future research will indeed consider exploring weakly-supervised or semi-supervised learning approaches to reduce the dependency on extensive manual annotations, thus lowering the overall cost and effort involved in dataset construction and labeling.

Second, SOY3DSEG does not yet support instance-level segmentation. The current model performs semantic segmentation on point clouds, labeling each point by class, but it cannot precisely delineate the boundaries of individual organs or objects. One potential future direction is to enable instance-level segmentation, allowing the model to identify and separate distinct plant organs (e.g., individual leaves, stems, fruits), and to enhance its capacity for fine-grained discrimination in real-world applications.

Further, although SOY3DSEG has shown strong cross-crop transferability, current validation efforts are primarily limited to seedling-stage datasets of soybean, maize, and tomato. There remains a lack of comprehensive evaluation across full growth cycles and on more structurally complex crops, such as climbing plants and fruit trees. Point cloud data from different crop types and developmental stages exhibit substantial variation in morphology, point density, and structural complexity. Future research should focus on validating the model using a broader range of crop datasets to rigorously assess its adaptability and generalization capacity across diverse agricultural scenarios.

Lastly, although SOY3DSEG has shown reliable performance from V1 to R7 growth stages in soybean, it does not yet handle edge cases such as pre-V1 seedling establishment, late senescence, or stress-affected morphologies. These conditions, such as overlapping foliage, adhered plant structures, and disease-induced morphological abnormalities, are common in real-world agricultural environments and can significantly impact model performance. These conditions, often characterized by low point densities, severe deformations, or atypical shapes, were not represented in the training and evaluation sets and may pose challenges to segmentation performance. Moreover, if the dataset contains point clouds with uneven distribution, there could be a decrease in segmentation accuracy, as sparse or unevenly distributed point clouds may not fully capture fine-scale features. Addressing these edge cases will require additional annotated datasets and methodological innovations, such as semi-/weak-supervised learning, synthetic data augmentation, temporal and multi-view fusion, and instance-aware or occlusion-aware sampling strategies.

In conclusion, although SOY3DSEG has demonstrated promising performance on existing crop point cloud datasets, future development should aim to reduce reliance on manual annotation, further improve segmentation accuracy, and enhance the model's adaptability across varying growth stages and crop types. These advancements will be critical for facilitating the broader application of 3D phenotyping and supporting the continued integration of precision agriculture technologies.

## 6 Conclusion

In the present study, we proposed SOY3DSEG, an efficient semantic segmentation model specifically designed for full-growth-cycle soybean point-cloud data. By integrating a Dynamic Multi-Stage Sampling Strategy (DMSS), a geometry-aware attention mechanism, and multi-scale feature fusion, SOY3DSEG demonstrates strong performance in soybean segmentation accuracy and in handling complex geometric structures. Experimental results show that SOY3DSEG maintains excellent stability throughout the soybean growth cycle and effectively processes point-cloud data from other crops such as maize and tomato, highlighting its cross-crop adaptability.

In addition to the network itself, this work provides a well-annotated full-growth-cycle soybean point cloud dataset together with a practical downsampling and feature-learning workflow. The proposed DMSS–SOY3DSEG pipeline is essentially backbone-agnostic and can be combined with other point-cloud architectures. We therefore expect the dataset and workflow to serve as a useful benchmark and reference for subsequent research on 3D crop phenotyping and semantic segmentation.

Moreover, the model significantly enhances segmentation accuracy across diverse growth stages, providing robust support for the advancement of precision agriculture technologies. This study offers novel perspectives and technical solutions for crop phenotyping, organ-level growth pattern analysis, and the evaluation of model generalization. Looking ahead, we plan to integrate the proposed dataset and downsampling strategy with more advanced backbone networks, such as Point Transformer v2, Point Transformer v3, and other recent Transformer-based or convolutional architectures, to build a more comprehensive benchmark on full-growth-cycle crop point clouds. SOY3DSEG and the accompanying dataset are expected to further contribute to the development of digital crop management, delivering more efficient and scalable solutions for crop monitoring and cultivation across a wide range of agricultural scenarios.

## Author contributions

J.N.: Data curation, methodology, and writing—original draft. J.T.: Conceptualization, investigation, and writing—review and editing. H.Z.: Methodology and software development. H.Zh.: Data analysis and validation. Z.Z.: Formal analysis and writing—review and editing. D.X.: Visualization and investigation. Q.C.: Supervision and project administration. R.Z.: Conceptualization, supervision, and writing—review and editing.

## Funding

This work was supported by the Research and Application of Key Technologies for Intelligent Farming Decision Platform (an Open Competition Project of Heilongjiang Province, China) under Grant No. 2021ZXJ05A03; the National Key R&D Program Project funded by the Ministry of Agriculture and Rural Affairs of the People's Republic of China under Grant No. 2025YFD2300401; the Programs for Science and Technology Development of Heilongjiang Province under Grant No. 2024ZX01A07; and the Heilongjiang Province Key Research and Development Plan (Innovation Bases), China under Grant No. JD2023GJ01.

## Data availability

The dataset and program code used in this study can be found at the link below: https://github.com/NiuJiarui718/SOY3DSEG.

## Declaration of competing interest

The authors declare that they have no known competing financial interests or personal relationships that could have appeared to influence the work reported in this paper.
