# Supplementary material for: SOY3DSEG: A high-precision universal point cloud segmentation model for soybean full growth period based on improved point transformer
Source: Plant Phenomics. 2026 Jan 10;8(1):100167. doi: 10.1016/j.plaphe.2026.100167 (PMC13109566; doi:10.1016/j.plaphe.2026.100167)
Supplement: Multimedia component 1 [file mmc1.docx]

**Supplementary Materials**

- 1. **3-D Point-Cloud Visualization of Key Developmental Stages in the Full Growth Cycle of Soybean**


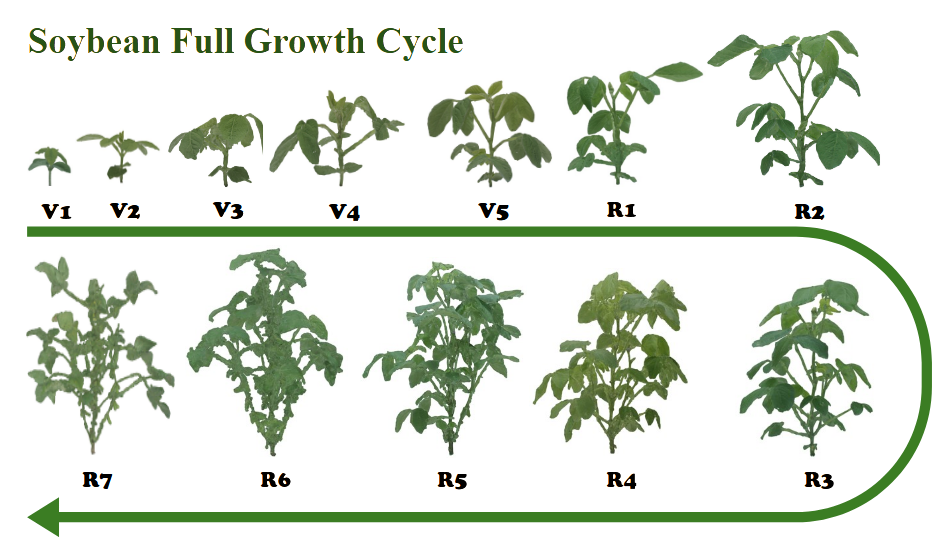


**Fig. S1.** Colorized 3-D point-cloud renderings of a single soybean plant at twelve key developmental stages (V1 – R7), derived from the Soybean Multi-View Stereo (Soybean-MVS) **point-cloud dataset**. **Top row:** vegetative phase — V1 (cotyledon expansion), V2 (first-node trifoliate), V3 (second-node trifoliate), V4 (third-node trifoliate), V5 (fourth-node trifoliate); early reproductive phase — R1 (begin bloom) and R2 (full bloom). **Bottom row:** reproductive phase — R3 (begin pod), R4 (full pod), R5 (begin seed), R6 (full seed), and R7 (physiological maturity). The sequence visually summarizes the progressive increase in canopy height, leaf area, and structural complexity that motivates a universal, full-season segmentation framework.

- 1. **DMSS algorithm implementation**

The following pseudocode outlines the steps involved in the reduced sampling process:

| **Algorithm** : Dynamic Adaptive Sampling Strategy |
| --- |
| 1. function downsample_point_cloud(input_folder, min_volume, max_volume, min_density, max_density, alpha, beta)  2. volumes ← [], densities ← []  3. for each file in input_folder do  4. if file ends with '.txt' do  5. points_df ← load_point_cloud(file)  6. points_tensor ← convert points_df to tensor  7. try:  8. volume, avg_density ← compute_metrics(points_tensor)  9. except exception:  10. continue  11. volumes.append(volume), densities.append(avg_density)  12. end  13. end  14. min_volume, max_volume ← volumes.min(), volumes.max()  15. min_density, max_density ← densities.min(), densities.max()  16. cv_volume ← std_volume / mean_volume, cv_density ← std_density / mean_density  17. total_cv ← cv_volume + cv_density  18. alpha, beta ← calculate_alpha_beta(cv_volume, cv_density, total_cv)  19. for each file in input_folder do  20. if file ends with '.txt' do  21. points_df ← load_point_cloud(file)  22. points_tensor ← convert points_df to tensor  23. sampling_ratio ← compute_sampling_ratio(volume, avg_density, min_volume, max_volume, min_density, max_density, alpha, beta)  24. sampled_points ← fps_sampling(points_tensor, num_points)  25. end  26. end  27. end function |

**Algorithm S1**. Pseudocode for DMSS

- 1. **Visual Comparison of Sampling Methods on Soybean Point Clouds at Different Growth Stages**


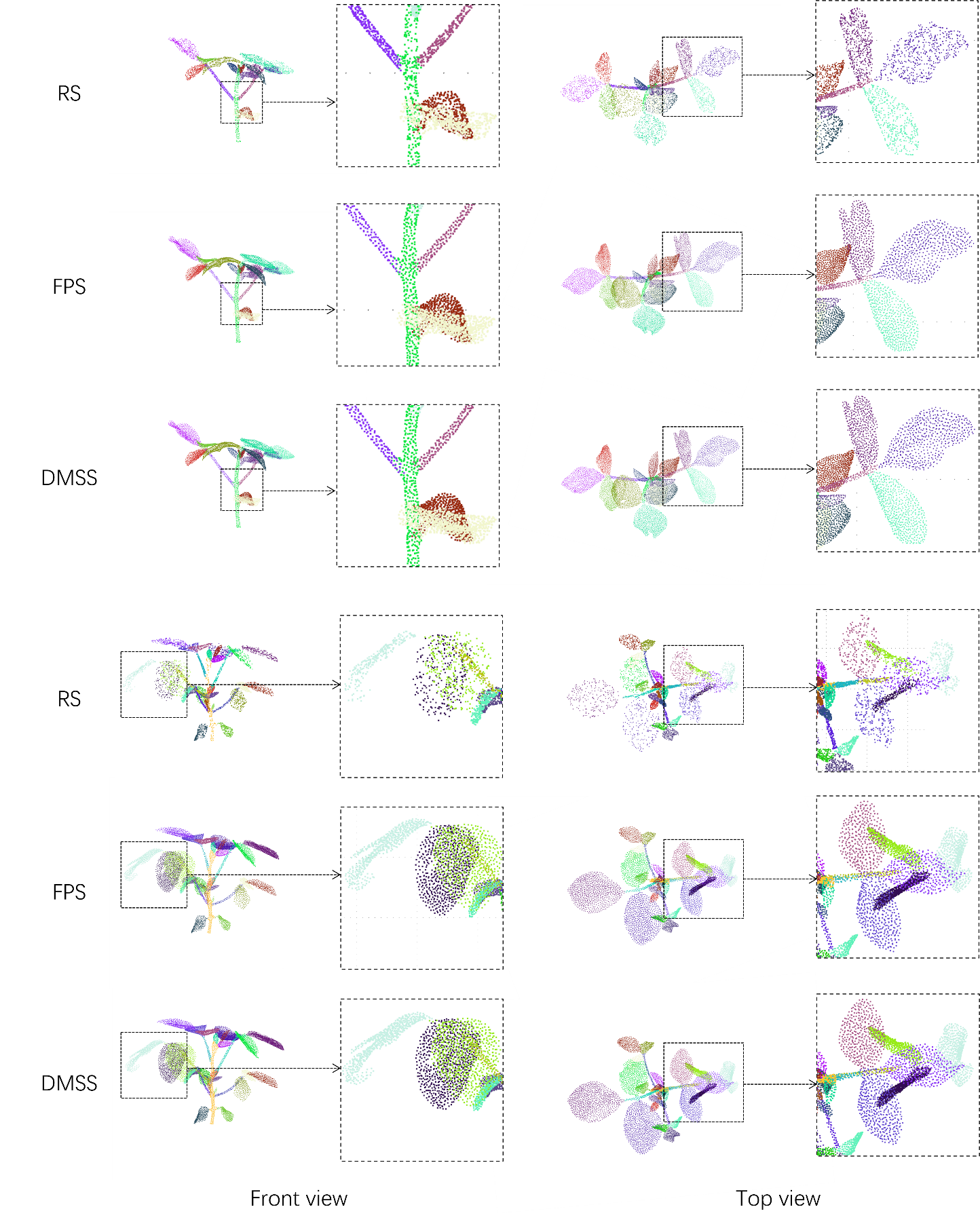


**Fig. S2.** Visual comparison of RS, FPS and DMSS on soybean point clouds at growth stages V2 and V5.


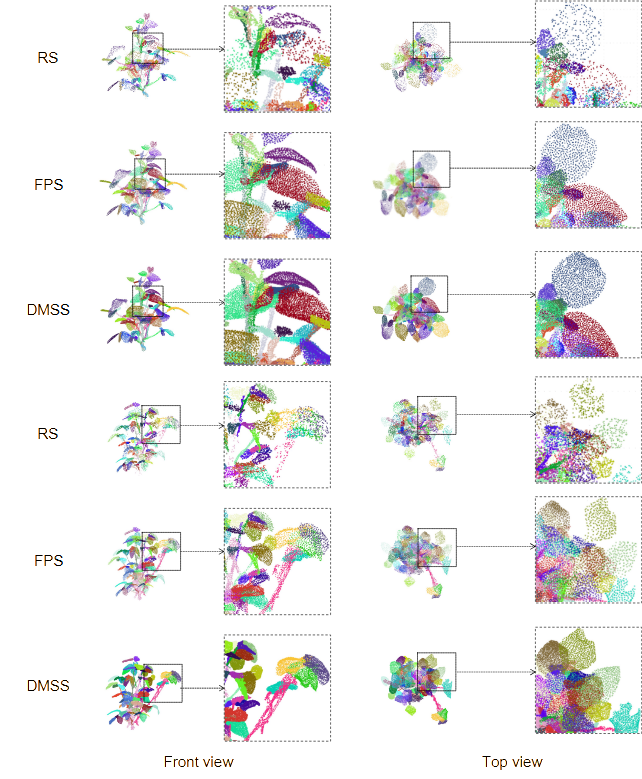


**Fig. S3.** Visual comparison of RS, FPS and DMSS on soybean point clouds at growth stages R4 and R6.

- 1. **Proportion of ISS Key Points Retained by Different Sampling Methods on Point Clouds During the Soybean Full Growth Cycle**

**Table. S1.** Different sampling methods retain the proportion of ISS key points for point clouds during the whole growth period.

| Point cloud\Sampling method | RS | FPS | DMSS |
| --- | --- | --- | --- |
| 20180612_DN251 | 0.7769 | 0.8469 | **0.8691** |
| 20180619_DN251 | 0.7683 | 0.8392 | **0.8584** |
| 20180626_DN251 | 0.7584 | 0.8269 | **0.8491** |
| 20180704_DN251 | 0.7491 | 0.8108 | **0.8397** |
| 20180711_DN251 | 0.7398 | 0.7905 | **0.8193** |
| 20180726_DN251 | 0.7195 | 0.7508 | **0.7986** |
| 20180817_DN251 | 0.6983 | 0.7502 | **0.7991** |
| 20180908_DN251 | 0.6992 | 0.7495 | **0.7498** |
| 20181003_DN251 | 0.7486 | 0.7583 | **0.7694** |

- 1. **Comparison of Sampling Times Across Soybean, Maize, and Tomato Datasets for Different Sampling Methods**

**Table. S2.** Comparison of sampling times

| Sampling Method\Dataset | Soybean | Maize | Tomato |
| --- | --- | --- | --- |
| RS | 146.21 | 12.42 | 33.17 |
| FPS | 3879.88 | 1844.64 | 2788.44 |
| DMSS | 2139.75 | 191.47 | 859.53 |

- 1. **The Average Accuracy of Each Model's Segmentation for DN251 During the Full Growth Period in 2019**

**Table. S3.** The average accuracy of each model segmentation of DN251 during the whole growth period

| **Growth period\Model** | **RandLA-Net** | **BAAF-Net** | **PointNet++** | **PointConv** | **PointTransformer** | **SOY3DSEG** |
| --- | --- | --- | --- | --- | --- | --- |
| V1 | 91.18 | 83.77 | 87 | 84 | 95.68 | **98.68** |
| V2 | 88.82 | 86.28 | 89 | 80 | 91.44 | **96.44** |
| V3 | 94.07 | 91.18 | 92 | 82 | 93.73 | **97.73** |
| V4 | 91.9 | 82.41 | 85 | 76 | 91.42 | **96.42** |
| V5 | 91.28 | 82.46 | 86 | 81 | 94.34 | **98.34** |
| R1 | 88.6 | 86.82 | 83 | 78 | 92.64 | **97.64** |
| R2 | 92.66 | 85.22 | 85 | 79 | 92.69 | **97.69** |
| R3 | 95.35 | 87.96 | 80 | 74 | 93.41 | **98.41** |
| R4 | 93.13 | 88.71 | 82 | 77 | 92.86 | **97.86** |
| R5 | 97.2 | 94.88 | 79 | 75 | 93.21 | **98.21** |
| R6 | 88.12 | 86.82 | 77 | 73 | 94.09 | **96.09** |
| R7 | 86.61 | 61.48 | 75 | 71 | 93.44 | **96.44** |

**Table. S4.**The average mIOU data of each model segmentation of DN251 during the whole growth period in 2019

| **Growth period\Model** | **RandLA-Net** | **BAAF-Net** | **PointNet++** | **PointConv** | **PointTransformer** | **SOY3DSEG** |
| --- | --- | --- | --- | --- | --- | --- |
| V1 | 67.58 | 54.2 | 26.36 | 40.6 | 83.45 | **94.74** |
| V2 | 69.15 | 56.76 | 27.47 | 41.99 | 84.22 | **93.61** |
| V3 | 71.57 | 60.04 | 29.55 | 44.11 | 84.76 | **94.84** |
| V4 | 70.77 | 57.55 | 28.03 | 42.42 | 84.03 | **93.63** |
| V5 | 69.66 | 58.35 | 29.28 | 43.01 | 82.09 | **94.29** |
| R1 | 67.91 | 59.71 | 29.99 | 42.83 | 81.16 | **94.03** |
| R2 | 70.33 | 58.17 | 28.82 | 42.86 | 80.53 | **94.20** |
| R3 | 73.56 | 60.32 | 27.64 | 40.7 | 82.77 | **93.94** |
| R4 | 72.01 | 61.57 | 26.85 | 41.68 | 81.06 | **93.59** |
| R5 | 74.4 | 64.82 | 25.41 | 39.75 | 80.34 | **91.70** |
| R6 | 68.55 | 58.97 | 24.85 | 39.18 | 77.68 | **92.26** |
| R7 | 66.64 | 52.11 | 23.4 | 38.15 | 78.91 | **89.26** |

- 1. **MIoU and mAccuracy Comparison Tables for Point Cloud Segmentation of Maize and Tomato at Seedling Stages**

**Table. S5.**mIoU and mAccuracy tables for maize point cloud segmentation

| **Time point** | **mIoU (%)** | **mAccuracy (%)** |
| --- | --- | --- |
| Day 1 | 99.8 | 99.9 |
| Day 3 | 99.7 | 99.9 |
| Day 5 | 99.2 | 99.6 |
| Day 7 | 99.1 | 99.8 |
| Day 10 | 98.7 | 99.5 |
| Day 13 | 98.5 | 99.3 |
| Day 15 | 98.9 | 99.5 |

**Table.S6.**mIoU and mAccuracy tables for tomato point cloud segmentation

| **Time point** | **mIoU (%)** | **mAccuracy (%)** |
| --- | --- | --- |
| Day 1 | 99.2 | 99.3 |
| Day 5 | 99.1 | 99.2 |
| Day 7 | 99 | 99.1 |
| Day 9 | 98.8 | 99 |
| Day 11 | 98.6 | 98.8 |
| Day 13 | 98.5 | 98.7 |
| Day 15 | 98.4 | 98.5 |
| Day 17 | 98.2 | 98.4 |
| Day 19 | 98 | 98.2 |

- 1. **Training dynamics (LR, Loss, mAcc, mIoU)**


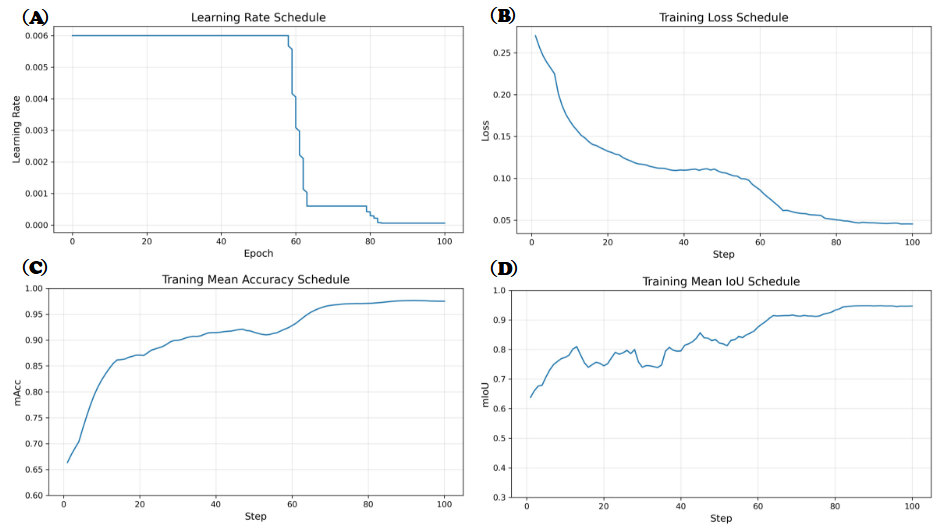


**Fig. S4.**  Training dynamics of the proposed model: (A) Learning rate schedule; (B) Training loss; (C) Training mean accuracy (mAcc); (D) Training mean Intersection-over-Union (mIoU).

- 1. **Model training time**

**Table. S7.**Training Duration of the Baseline Model and SOY3DSEG for Different Crops (Per 100 Batches, Unit: min).

| **Crops/Models** | Soybean | Maize | Tomato |
| --- | --- | --- | --- |
| Baseline | 643 | 56 | 78 |
| SOY3DSEG | 622 | 54 | 77 |
